# Rare incidence of cholecystocolonic fistula and robotic-assisted surgical management

**DOI:** 10.1016/j.ijscr.2021.105770

**Published:** 2021-03-17

**Authors:** M. Bilal, A. Lim, B. Tuai, J. Eisner, Janet L. Hobbs

**Affiliations:** a Graduate Medical Education, General Surgery Residency Program, Community Memorial Health System, Ventura, CA, USA; b Community Memorial Health System, Ventura, CA, USA; c Graduate Medical Education, Community Memorial Health System, Ventura, CA, USA

**Keywords:** Case report, Cholecystocolonic fistulas, Robotic surgery

## Abstract

We present a patient who was found to have a cholecystocolonic fistula during robotic cholecystectomy. The patient initially presented with Non-ST-elevation myocardial infarction (NSTEMI) and biliary obstruction. A delayed cholecystectomy was performed robotically after managing his Coronary Artery Disease (CAD) and relief of his biliary obstruction with endoscopic retrograde cholangiopancreatography (ERCP). Intraoperatively, a cholecystocolonic fistula, was found. This case report aims to highlight intraoperative management of the fistula and review the existing literature.

## Highlights

• This case report aims to highlight intraoperative management of the fistula and review the existing literature.
• Cholecystocolonic fistulas are one of the rare complications associated with gallstone disease.
• Close evaluation of patient imaging studies and clinical presentation is of utmost importance.

## 1 Introduction

Biliary fistulas to various structures have been reported in the literature. They can connect the gallbladder with the biliary tree and rarely involve the gastrointestinal tract (internal fistulas) and the abdominal wall (external fistulas) [1]. Although rare, the etiology is related to chronic inflammation and erosion of the gallstones due to pressure onto nearby structures, including but not limited to, stomach, duodenum, or colon. The cholecystocolonic fistula is an uncommon but pertinent complication of gallbladder disease, occurring in 0.06%–0.14% of patients with the biliary disease [2]. Failure to identity these fistulas intraoperatively can lead to morbidity following surgery resulting in colonic perforation, peritonitis, or death.

## 2 Case presentation

**Fig. 1:**
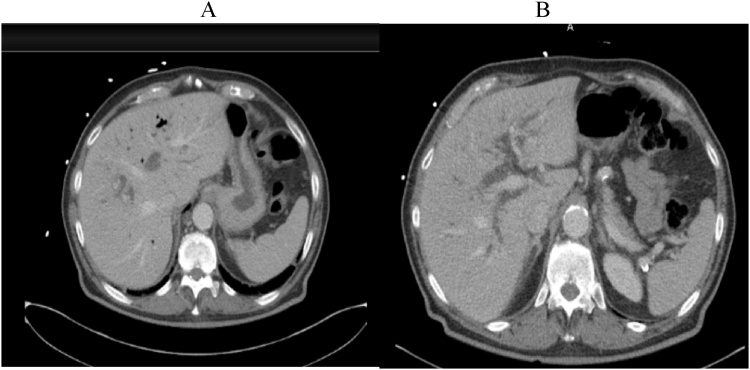
Image A. Pneumobilia; Image B. Intrahepatic ductal dilation.

The patient is a 79-year-old male with a past medical history of hypertension who presented to the emergency department with an ongoing history of weakness, fatigue malaise, and right upper quadrant abdominal pain. Labs showed transaminitis as well as elevated bilirubin concerning biliary obstruction. The patient was also noted to have gram-negative rod bacteremia. Computerized Tomography (CT) scan of the abdomen pelvis revealed significant intra and extrahepatic ductal dilation and intra and extrahepatic pneumobilia (Fig. 1). He underwent an ERCP and found to have thick sludge and stone debris from the ampullary orifice. Due to a concern for a possible filling defect in the distal common bile duct versus possible stricture, distal common bile duct brushings were performed in addition to biopsies and stenting with a 10 French 5 cm biliary stent. Pathology from the brushing and biopsies were negative for dysplasia or malignancy.

**Fig. 2:**
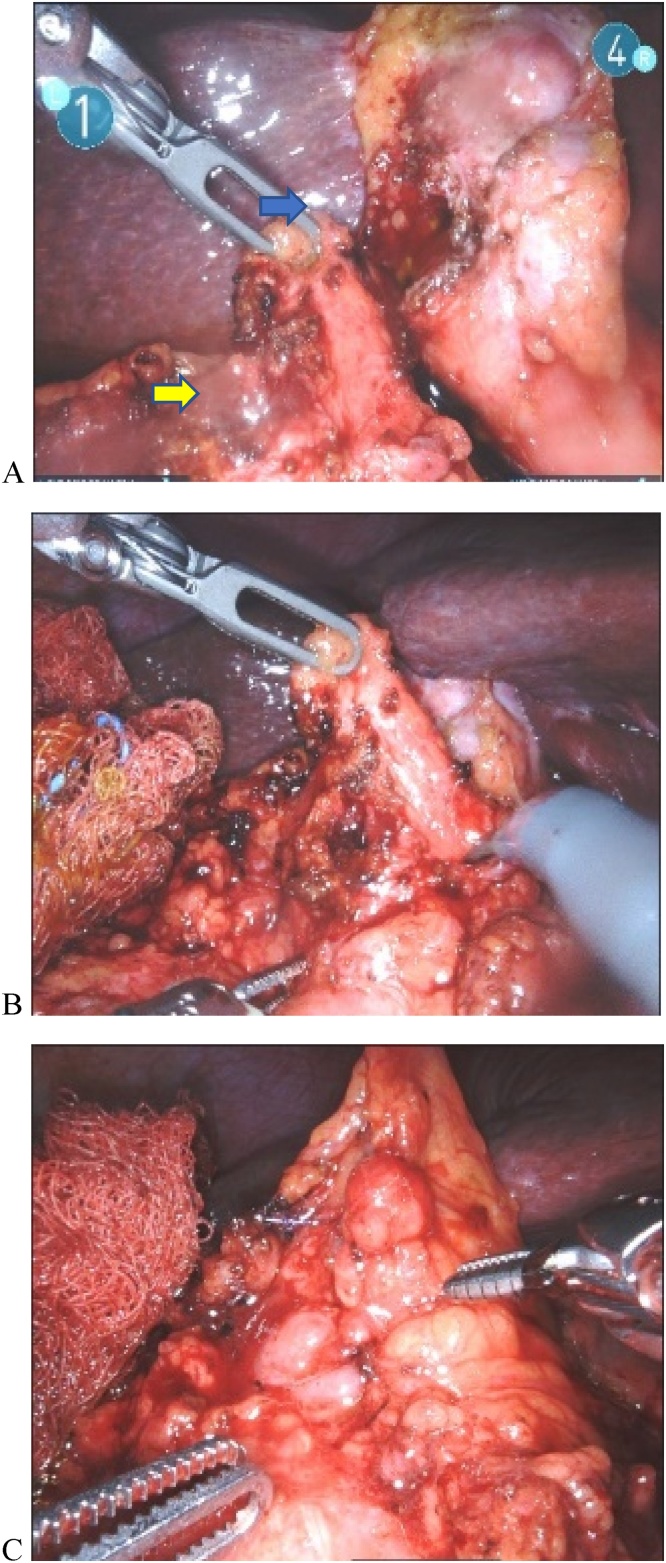
Image A. Blue arrow shows opening in the gallbladder and yellow arrow shows opening in the transverse colon; Image B. Shows colonic opening; Image C. Shows two-layer closure bowel.

During his hospitalization, the patient was found to have elevated troponins and electrocardiogram (EKG) changes consistent with NSTEMI. He underwent a pharmacological stress test followed by coronary angiography. He was found to have chronic occlusion of his Right Coronary Artery (RCA). Given these findings and acute coronary events, surgical intervention for cholecystectomy was delayed. The patient was brought back for robotic cholecystectomy after subsequent follow-up and clearance with his cardiologist and surgery clinic. Intraoperative findings revealed dense adhesions to the gallbladder involving the omentum and colon. A dense adhesion was present between the gallbladder and the transverse colon, and a provisional diagnosis of cholecystocolonic fistula was made on further dissection. The case was continued robotically, and the diagnosis of cholecystocolonic fistula was confirmed with the finding of a fistula between the fundus of the gallbladder and the transverse colon (Fig. 2). The fistula was divided. The gallbladder dissected in dome down method. The cystic artery and ducts identified and ligated, and the gallbladder was removed. The colon fistula site was 5 mm in size and was primarily repaired with two layers using a V-lock suture.

## 3 Discussion

Cholecystocolonic fistulas is one of the rare complications associated with gallstone disease. Etiology is related to the pressure effect and inflammation of the biliary system onto adjacent organs. The management of cholecystocolonic fistulas is varied based on the literature because of the low incidence of cases [3]. Regardless of the presence or absence of symptoms, this condition has been treated with fistula resection and cholecystectomy. If necessary, a common bile duct exploration is performed [4]. Based on the literature, surgical management is preferred because it theoretically corrects the fistula and removes the risk of sepsis from bacterial translocation [2]. Mortality is found to be as high as 13% in cases related to biliary sepsis [5,6]. Another aspect of management amongst authors is the one-stage versus two-stage operation. The one stage process entails completing the intervention all in the same setting. Whereas the two-stage process include, the fistula's transection with a repair of the fistula and a delayed cholecystectomy [7]. The stages of operation is a technical and dynamic process that is influenced by various factors, such as patient comorbidities, technical difficulty, and clinical conditions.

## 4 Conclusion

The diagnosis of cholecystocolonic fistula is a rare incidence related to chronic cholecystitis. In these cases, a closer evaluation of patient imaging studies and clinical presentation is of utmost importance. The choice of surgical intervention, lap vs. open and now robotic surgery, is determined by the patient’s clinical condition, experience, and the safest outcomes.

## Declaration of Competing Interest

All authors don’t have any financial and personal relationships with other people or organisations to disclose.

## Funding

Not applicable.

## Ethical approval

A single case reports application was submitted to IRB. A draft of the case report was evaluated by members of the research team to ascertain that the manuscript was de-identified.

## Consent

A patient consent was obtained.

## Author contribution

Study concept, design, and interpretation: B. Tuai MD, J. Eisner MD.

Writing the paper: M. Bilal DO, A. Lim MD.

## Registration of research studies

Not Applicable.

## Guarantor

J. Eisner MD.

## Provenance and peer review

Not commissioned, externally peer-reviewed.
